# Supplementary material for: TLR4-Mediated Recognition of Mouse Polyomavirus Promotes Cancer-Associated Fibroblast-Like Phenotype and Cell Invasiveness
Source: Cancers (Basel). 2021 Apr 25;13(9):2076. doi: 10.3390/cancers13092076 (PMC8123340; doi:10.3390/cancers13092076)
Supplement: Supplementary file 1 [file cancers-13-02076-s001.zip › cancers-1155502-supplementary.pdf]

Figure S1

Un-cropped images for Figure 3c

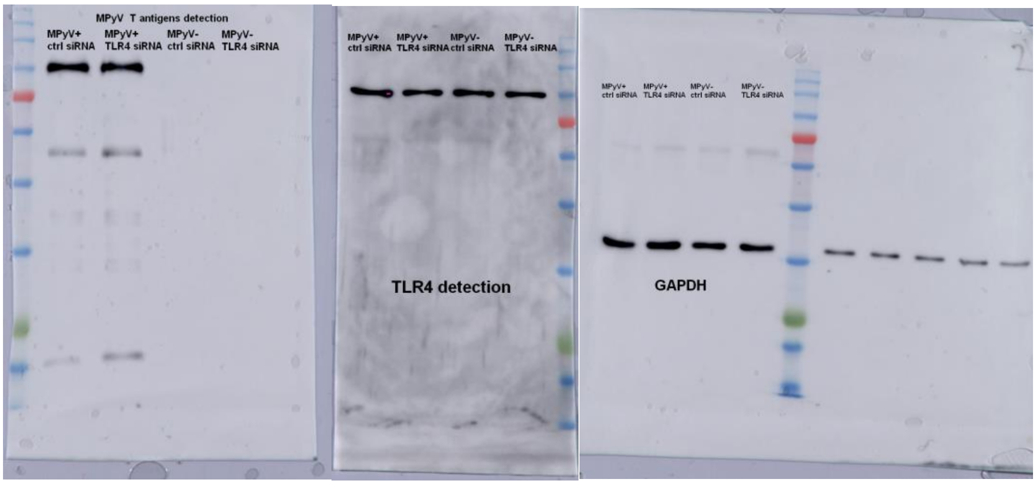

Un-cropped images for Figure 4b

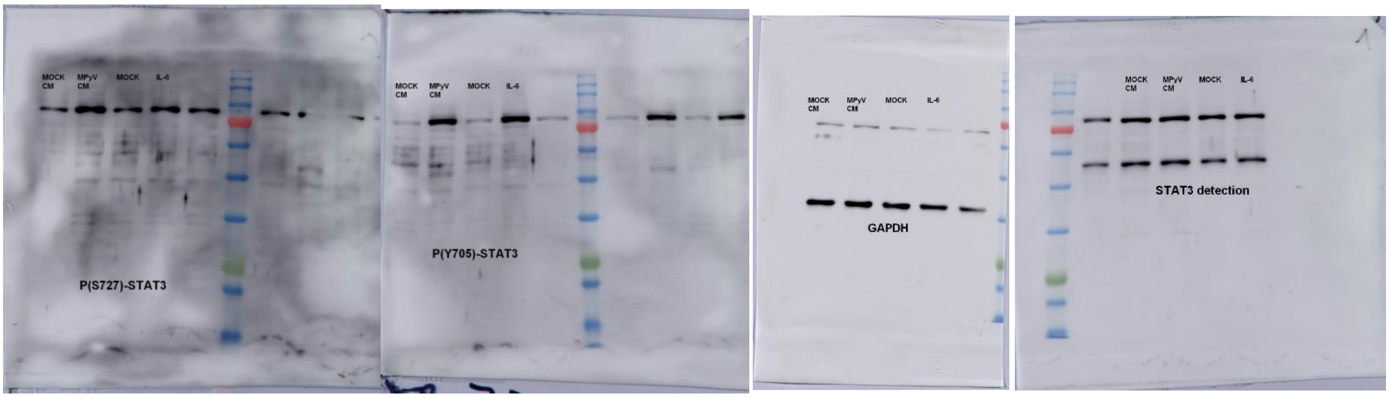

Un-cropped images for Figure 4c

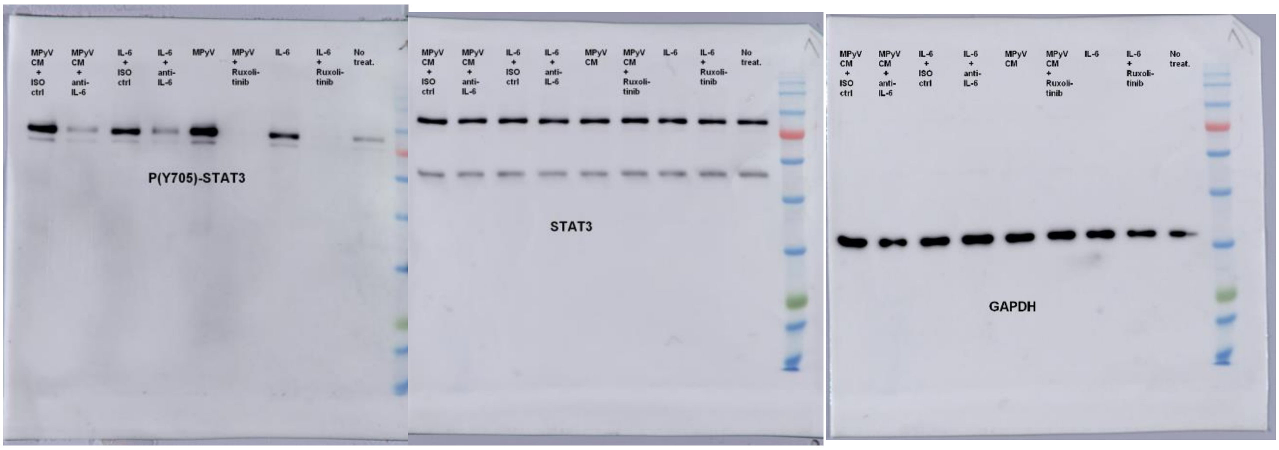

Un-cropped images for Figure 5a

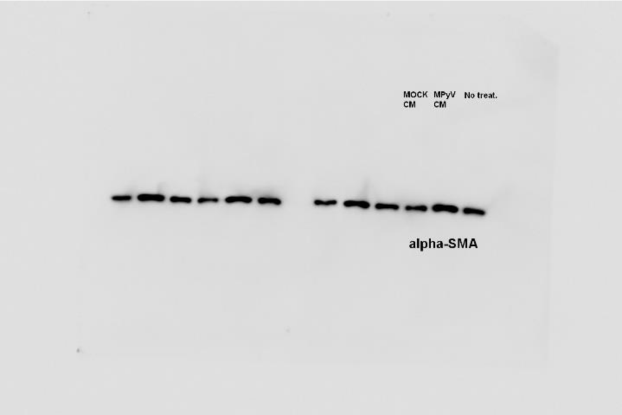

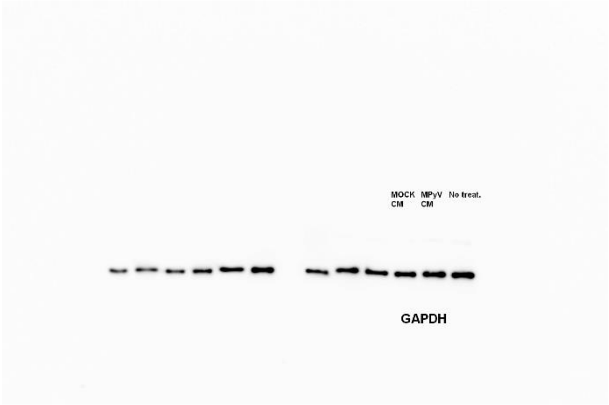

Un-cropped image for Figure S3a

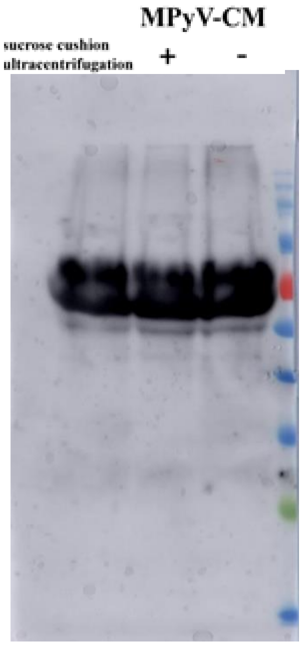

Figure S2

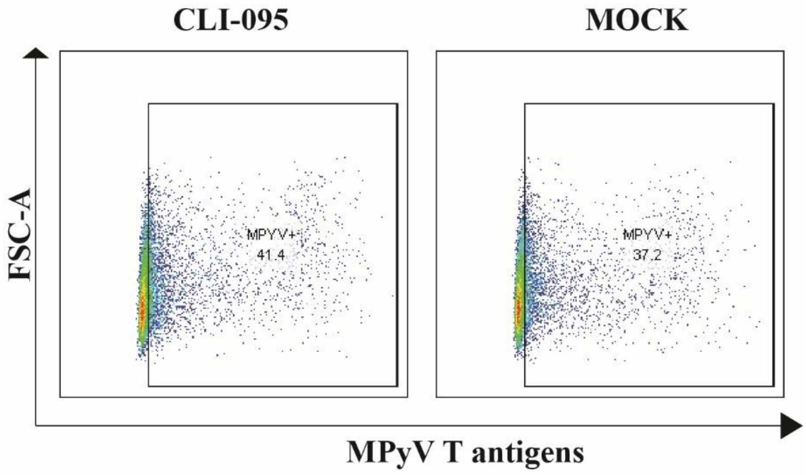

Figure S2. Effect of TLR4 signaling on MPyV lifecycle in 3T6 cells. Flow cytometry analysis of the percentage of MPyV positive MEFs determined by expression of T antigens in the presence or absence (MOCK) of TRL4 inhibitor CLI-095 (10 μM).

Figure S3

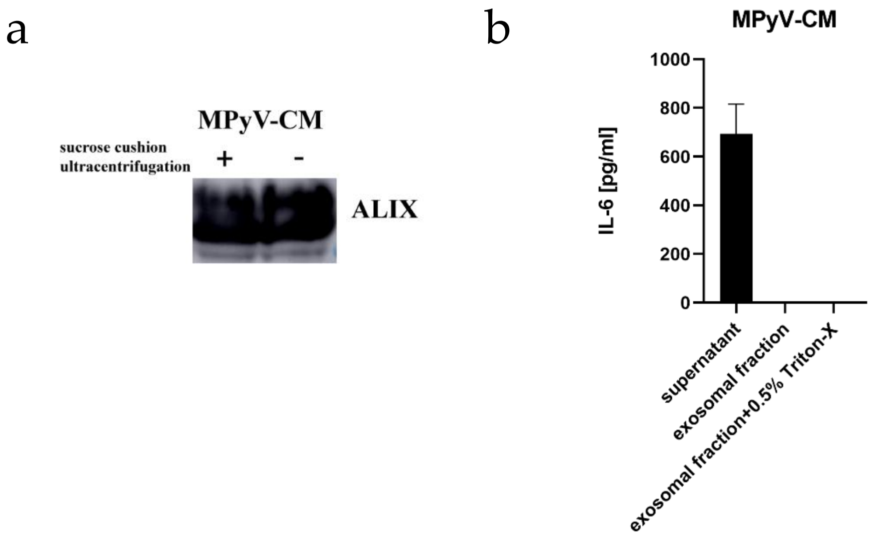

Figure S3. Analysis of MPyV-CM. (a) The detection of exosomal marker ALIX in exosomal fraction from MPyV-CM. (b) The presence of soluble IL-6 and exosomal IL-6 determined by ELISA.
